# Swallowing After Open Partial Horizontal Laryngectomy Type IIa: A Quantitative Videofluoroscopic Analysis Using the Analysis of Swallowing, Physiology, Events, Kinematics, and Timing

**DOI:** 10.1044/2026_JSLHR-25-00521

**Published:** 2026-06-10

**Authors:** Raphaela da Costa Miranda Barbosa, Andressa Silva de Freitas, Rayane Beltrão Alves Cerqueira, Fernando Luiz Dias, Renata Mancopes, Catriona M. Steele

**Affiliations:** a National Cancer Institute - INCA, Rio de Janeiro, Brazil; b KITE Research Institute – University Health Network, Toronto, Ontario, Canada; c Rehabilitation Sciences Institute, Temerty Faculty of Medicine, University of Toronto, Ontario, Canada; d Canada Research Chair in Swallowing & Food Oral Processing, Ottawa, Ontario, Canada

## Abstract

**Purpose:**

The purpose of this study was to characterize swallowing physiology on sips of thin liquid in patients following open partial horizontal laryngectomy Type IIa (OPHLIIa) using videofluoroscopic swallow study (VFSS) data, rated using the Analysis of Swallowing Physiology: Events, Kinematics, and Timing (ASPEKT) method.

**Method:**

This retrospective study involved secondary analysis of archived data for a cross-sectional sample of 100 patients (94 men, six women; *M*_age_ = 67 years) who underwent VFSS after OPHLIIa surgery at the Brazilian National Cancer Institute (mean time postsurgery = 44 months). For each patient, the first available comfortable sip of thin liquid barium was rated using ASPEKT parameters and additional hypopharyngeal area and cricopharyngeal tissue redundancy measures. Ratings were performed by trained speech-language pathologists using ImageJ software. Values were compared to reference data for adults without dysphagia using *t* tests and odds ratio analysis.

**Results:**

After OPHLIIa surgery, patients in this sample exhibited elevated frequencies and significantly increased odds of atypical findings across multiple swallowing parameters. With respect to impaired swallowing safety and efficiency, these included clinically significant Penetration–Aspiration Scale scores (22%), partial/incomplete laryngeal vestibule closure (LVC; 34%), and increased pharyngeal residue, both overall (76%) and by subspace: valleculae (49%), pyriform sinuses (63%), and elsewhere in the pharynx (57%). Other parameters showing significantly increased odds of atypical findings included reduced pharyngeal constriction (98%), reduced hyoid peak position (83%) and speed (59%), a prolonged hyoid-burst-to-upper-esophageal-sphincter-opening interval (45%), and the presence of redundant cricopharyngeal muscle tissue (39%). The analyses highlighted poor hypopharyngeal constriction as a feature of swallowing post-OPHLIIa and revealed apparent compensations in the form of significantly shorter time-to-LVC and prolonged LVC and upper esophageal sphincter opening durations.

**Conclusions:**

Swallowing dysfunction following OPHLIIa is marked by structural and physiological changes that impair both swallowing safety and efficiency. These findings highlight critical physiological impairments, particularly reduced pharyngeal constriction and compromised airway protection, which should be considered during clinical evaluation and decision making.

Laryngeal cancer is the most frequent head and neck cancer, accounting for 60%–65% of cases (National Comprehensive Cancer Network, 2024). According to projections from the GLOBOCAN *Cancer Tomorrow* tool, the estimated number of new larynx cancer cases worldwide from 2022 to 2025 is 200,606. During the same period, the estimated number of deaths from larynx cancer is 109,928(International Agency for Research on Cancer, 2022). Treatment approaches are determined by disease extent and patient-specific factors, often involving surgery, chemoradiation, or both (Botti et al., 2024; National Comprehensive Cancer Network, 2024). Surgical options include conservative or radical procedures, such as partial laryngectomies, designed to achieve a good local control rate and preserve patients' swallowing, breathing, and voice function (Liu et al., 2024; Sperry et al., 2013). Open partial horizontal laryngectomies (OPHLs), also known as supracricoid partial laryngectomy, have become an important option in modern surgical practices aimed at preserving laryngeal function. Over the past few decades, these procedures have evolved and shown excellent outcomes in treating intermediate and locally advanced laryngeal cancer (de Vincentiis et al., 2022; Steuer et al., 2017). Among OPHL approaches, Type IIa or cricohyoidoepiglottopexy (CHEP) is a promising choice for managing laryngeal T1b to T4a tumors. CHEP offers effective oncological control while allowing most patients to resume oral intake without reliance on a feeding tube and to achieve decannulation, in contrast to the permanent stoma associated with total laryngectomy. The procedure involves the removal of multiple anatomical structures, including the pre-epiglottic space, epiglottic pedicle, thyroid cartilage, laryngeal ventricles, vocal folds, vestibular folds, and paraglottic space. Depending on the case, either one or both arytenoids are preserved. However, this procedure results in the loss of key protective structures in the lower airway, significantly impacting swallowing biomechanics (H. Laccourreye et al., 1990; Schindler, Pizzorni, Fantini, et al., 2016; Schindler, Pizzorni, Mozzanica, et al., 2016). Previous studies describing swallowing after OPHL Type IIa (OPHLIIa) have predominantly described the extent to which functional swallowing of unrestricted diets has been achieved within 1 year postsurgery (Adamopoulos et al., 2000; Bron et al., 2000; Crampette et al., 1999; de Vincentiis et al., 2022; Kelchner & Klaben, 2013; Goeleven et al., 2005; H. Laccourreye, 1990; O. Laccourreye et al., 1998; Lima et al., 2001). Several studies involving flexible endoscopic examinations point to neoglottic incompetence and aspiration (typically silent) being a primary postoperative concern, particularly for cases involving arytenoid resection (Alicandri-Ciufelli et al., 2013; Liu et al., 2024; Schindler, Pizzorni, Fantini, et al., 2016; Schindler, Pizzorni, Mozzanica, et al., 2016; Woisard et al., 1996; Zacharek et al., 2001). Schindler, Pizzorni, Fantini, et al. (2016) also mention residue as a concern on all consistencies. Three prior studies involving videofluoroscopic swallow study (VFSS) provide greater detail regarding altered swallowing physiology in this population. Lewin et al. (2008) reported aspiration as a continuing concern in 70% of OPHLIIa patients at a follow-up VFSS performed, on average, 7 weeks postsurgery. Importantly, compensatory strategies were effective for mitigating aspiration in the majority of patients. Additional observations in their study, which included both OPHLIIa and OPHL Type IIIa (cricohyoidopexy) patients, included reduced hyolaryngeal excursion (45%), reduced tongue base retraction toward the posterior pharyngeal wall (27%), and residue (44%). A more recent article by Pizzorni et al. (2019) reported more detail regarding VFSS measures in patients following OPHLIIa with arytenoid resection. Impaired laryngeal closure was identified as the primary mechanism underlying unsafe swallowing, seen in 47% of their sample. Inefficiency, seen in 40%, was associated with prolonger pharyngeal transit time, reduced maximum distention of the upper esophageal sphincter (UES), and poor tongue base retraction. Freitas et al. (2022) reported high rates of penetration (67%) and aspiration (34%) in a sample of 70 OPHLIIa patients and found statistically significant associations between postswallow residue (particularly on the tongue base) and penetration–aspiration.

The purpose of this study was to build on this prior literature characterizing changes to swallowing function and physiology after OPHLIIa using a detailed analysis of thin liquid swallows from lateral-view VFSS exams. In particular, we aimed to use an analysis approach known as the Analysis of Swallowing Physiology: Events, Kinematics, and Timing (ASPEKT) method (Steele et al., 2023) to describe both the frequency and extent to which values for quantitative VFSS parameters in OPHLIIa patients fall outside the typical range seen for liquid swallowing in adults without dysphagia. Prior research by Steele et al. (2023), in a sample of 78 healthy adults (aged 21–82 years), established reference interval statistics (median and 2.5th and 97.5th percentiles) for 17 different quantitative VFSS parameters on comfortable sips of thin liquid as well as comfortable sips of slightly thick and mildly thick liquid and teaspoon boluses of moderately and extremely thick liquid, as defined by the International Dysphagia Diet Standardisation Initiative (IDDSI; Cichero et al., 2017). Additionally, they proposed a definition of “typical values” (i.e., values of no clinical concern) as values falling in the healthy interquartile range (IQR) and clinical decision limits for differentiating values of potential clinical concern (i.e., “atypical values”) set at either the 25th or 75th percentile values of the healthy reference range, depending on the directionality of each parameter. Since the original introduction of the ASPEKT method (Steele et al., 2019), this approach has been used to describe both the frequency of atypical values and the degree to which descriptive statistics (median and IQR) differ from those seen in healthy adults in patients from various etiological groups, including those with chronic obstructive pulmonary disease (COPD; Mancopes et al., 2020), amyotrophic lateral sclerosis (Gandhi et al., 2024; Waito et al., 2020), oropharynx cancer treated with radiation (Barbon et al., 2021), traumatic spinal cord injury (Valenzano et al., 2023), and Parkinson disease (Gandhi et al., 2021). Analyses using the ASPEKT method have also been effective for identifying VFSS parameters with high frequencies of atypical values (i.e., > 25%) that may act as hallmarks of dysphagia in specific etiological groups, such as the combination of poor pharyngeal constriction and shortened UES opening (UESO) duration in patients with COPD (Mancopes et al., 2020). Our group previously published a preliminary analysis using the ASPEKT method to characterize swallowing in 10 patients following OPHLIIa surgery (Barbosa et al., 2024). In that sample, two patients showed incomplete laryngeal vestibule closure (LVC) with associated penetration, while the majority of the sample showed functional airway protection without penetration–aspiration but prolonged latencies to LVC. Residue was a prominent finding associated with poor pharyngeal constriction, with reduced or absent constriction of the hypopharynx noted as a particular characteristic of the sample. Apparent compensations in the form of prolonged LVC and UESO durations were also observed. Building on that foundation, the current study applies the ASPEKT method to analyze archived VFSS recordings from a substantially larger sample of 100 patients who underwent OPHLIIa surgery, aiming to determine whether features described in the previous preliminary study are confirmed in a larger sample and to provide a more comprehensive understanding of postoperative swallowing outcomes.

## Method

### Participants

We conducted a retrospective analysis of VFSS recordings from a cross-sectional sample of patients who had undergone OPHLIIa surgery at the Head and Neck Cancer Surgery Section of the Brazilian National Cancer Institute (Instituto Nacional de Câncer [INCA]) prior to 2018 and for whom a VFSS recording of a swallowing with a comfortable sip of thin liquid was available. These individuals had previously provided written informed consent for inclusion of their sociodemographic and videofluoroscopy recordings in a research database under Research Ethics Committee of the National Cancer Institute (INCA; Certificado de Apresentação de Apreciação Ética [CAAE]: 26331314.2.0000.5274). For the current study, we extracted cases from the database for 100 patients over 18 years of age, without active disease, who had undergone OPHLIIa, with no staging restriction. Patients who had undergone other surgeries in the head and neck region before or after the OPHLIIa were excluded.

### VFSS Data and Rating Process

We performed secondary analysis of lateral view VFSS recordings of thin liquid swallows that were in the research database; these procedures were performed beginning in 2011. These VFSSs were previously collected during clinical care using continuous fluoroscopy at 30 frames per second. It is important to clarify that VFSS was not performed routinely for every patient postsurgery; rather, it was conducted either as part of a follow-up protocol or when patients reported swallowing difficulties. The number, consistencies, and volumes of stimuli in these archives VFSS exams varied across patients. The only task that was available in all recordings, and which overlapped with the tasks used by Steele et al. (2023) for the development of ASPEKT reference values, was a comfortable sip of thin liquid barium. Therefore, the first thin liquid sip for each patient was extracted for analysis. The thin liquid stimulus was Bariogel barium diluted to a 50% weight-to-volume concentration with water, which has subsequently been verified to meet the IDDSI definition of thin liquid consistency via the IDDSI Flow Test (Hanson et al., 2019). Patients were instructed to hold the bolus in their mouth and swallow upon the investigator's command.

Speech-language pathologists trained in the ASPEKT method (Steele et al., 2023) performed independent duplicate ratings of each video recording using ImageJ software (ImageJ Version 1.54g; National Institutes of Health, https://imagej.nih.gov). Rating occurred in two stages. During the first stage, raters determined the number of swallows per bolus, Penetration–Aspiration Scale (PAS; Rosenbek et al., 1996) scores for each swallow, and the frame numbers of key events in the swallowing sequence. Of note, removal of the false and true vocal folds, and in some cases of one arytenoid during OPHLIIa surgery, eliminates anatomical landmarks that are used in scoring the depth of airway invasion using the PAS. As shown in Table 1, definitions for the different levels on the PAS were made to reflect anatomical changes arising from OPHLIIa surgery. Specifically, the inferior border of the remaining arytenoid cartilage(s) was used as a proxy landmark for vocal fold location to distinguish scores of 4 and 5 from both higher and lower levels of airway entry. After confirming the frames of interest for key events, these frames were used in the second stage of rating, where pixel-based measures of structural movement or area were taken and normalized to an anatomical scalar, that is, the length of the C2–C4 vertebral spine (Molfenter & Steele, 2014). Duplicate ratings were reviewed for agreement, with discrepancies resolved by consensus. Preconsensus interrater agreement was assessed by calculating the median and IQR range for the absolute difference in ratings across raters for each clip. Cohen's kappa scores were used to measure agreement for categorical parameters, and two-way mixed-model intraclass correlation coefficients (ICCs) were calculated for event identification and pixel-based measures.

**Table 1.** Adaptation of the Penetration–Aspiration Scale for use with open partial horizontal laryngectomy Type IIa (OPHLIIa) patients.

Score	Penetration–Aspiration Scale	Adaptation for OPHLIIa (absence of true vocal folds)
1	Material does not enter the airway.	No adaptation required; bolus remains entirely outside the laryngeal vestibule.
2	Material enters the airway, remains above the vocal folds, and is ejected.	Material enters the airway, remaining above the inferior margin of the remaining arytenoid cartilage(s), and is ejected.
3	Material enters the airway, remains above the vocal folds, and is not ejected.	Material enters the airway, remaining above the inferior margin of the remaining arytenoid cartilage(s), and is not ejected.
4	Material enters the airway, contacts the vocal folds, and is ejected.	Material enters the airway, reaches the level of the inferior margin of the remaining arytenoid cartilage(s), (used as a proxy landmark for the level of the vocal folds), and is ejected.
5	Material enters the airway, contacts the vocal folds, and is not ejected.	Material enters the airway, reaches the level of the inferior margin of the remaining arytenoid cartilage(s), (used as a proxy landmark for the level of the vocal folds), and is not ejected.
6	Material enters the airway, passes below the vocal folds, and is ejected.	Material enters the airway, passes below level of the inferior margin of the remaining arytenoid cartilage(s), and is ejected.
7	Material enters the airway, passes below the vocal folds, and is not ejected despite effort.	Material enters the airway, passes below the level of the inferior margin of the remaining arytenoid cartilage(s), and is not ejected despite effort.
8	Material enters the airway and passes below the vocal folds, and no effort is made to eject.	Material enters the airway and passes below the level of the inferior margin of the remaining arytenoid cartilage(s), and no effort is made to eject.

Definitions for the parameters of interest are provided in Table 2. Parameters were classified as typical or atypical by comparing them to the clinical decision limits proposed by Steele et al. (2023), as follows:

- PAS scores were dichotomized into scores of no clinical concern (1, 2, or 4), for which any penetrated material has been ejected from the laryngeal vestibule by the end of the swallow, versus clinically significant scores (3, 5, 6, 7, or 8) as proposed previously by Steele and Grace-Martin (2017).
- LVC integrity ratings were dichotomized as typical (complete) or atypical (partial or incomplete).
- Continuous parameters for which smaller values would be indicative of potential clinical concern (e.g., short durations of LVC or UESO) were classified as atypical if they fell below the 25th percentile of the healthy reference distribution.
- Continuous parameters for which larger values would be indicative of potential clinical concern (e.g., excessive pharyngeal residue) were deemed atypical if they fell above the 75th percentile of the healthy reference distribution.

**Table 2.** Analysis of Swallowing Physiology: Events, Kinematics, and Timing method parameters measured in this study.

Parameter	Metric	Definition
Penetration–Aspiration Scale	Categorical	8-point scale characterizing depth of and response to penetration–aspiration (Rosenbek et al., 1996)
LVC integrity	Categorical	Complete, partial, or incomplete closure
Swallow reaction time	Milliseconds	Interval from bolus passing mandible (the first frame where the leading edge of the bolus touches or crosses the shadow of the inferior ramus of the mandible) until HYB (the first frame of a superior or superior/anterior jump in hyoid position with a larger magnitude of displacement across the two frames than in preceding position changes)
Hyoid burst onset to UES opening	Milliseconds	Interval from HYB until UES opening
UES opening duration	Milliseconds	Interval from UES opening until UES closure
Time-to-LVC	Milliseconds	Interval from HYB until the first frame of most-complete LVC (the first frame showing the smallest space in the laryngeal vestibule, anterior to arytenoid cartilages, and inferior to the epiglottis)
LVC duration	Milliseconds	Interval between the first frame of most-complete LVC and LVC offset
Peak XY hyoid position	%(C2–C4)	Distance along the hypotenuse between the anterior inferior corner of the hyoid bone and the anterior inferior corner of C4 on the frame of peak anterior–superior hyoid position, normalized to the length of the C2–C4 vertebral spine
Hyoid XY speed	%(C2–C4)/second	Change in XY hyoid position divided by movement duration from the HYB frame to the frame of peak XY hyoid position, normalized to the length of the C2–C4 vertebral spine
Maximum UES distention	%(C2–C4)	Pixel-based measure of lateral view UES opening on the frame of maximum UES opening, normalized to the length of the C2–C4 vertebral spine
Pharyngeal area at rest	%(C2–C4)^2^	Pixel-based measure of the two-dimensional lateral area of the pharynx on the frame of swallow rest, normalized to the squared length of the C2–C4 vertebral spine
Pharyngeal area at maximum pharyngeal constriction	%(C2–C4)^2^	Pixel-based measure of the two-dimensional lateral area of the unobliterated pharynx on the frame of maximum pharyngeal constriction
Vallecular residue	%(C2–C4)^2^	Pixel-based measure of the two-dimensional lateral area of residue in the vallecular space on the swallow rest frame, normalized to the squared length of the C2–C4 vertebral spine
Pyriform residue	%(C2–C4)^2^	Pixel-based measure of the two-dimensional lateral area of residue in the pyriform sinuses on the swallow rest frame, normalized to the squared length of the C2–C4 vertebral spine
Other pharyngeal residue	%(C2–C4)^2^	Pixel-based measure of the two-dimensional lateral area of residue elsewhere in the pharynx on the swallow rest frame, normalized to the squared length of the C2–C4 vertebral spine
Total pharyngeal residue	%(C2–C4)^2^	Sum of residue measures for the valleculae, pyriform sinuses, and elsewhere in the pharynx on the swallow rest frame

*Note.* LVC = laryngeal vestibule closure; HYB = hyoid burst onset; UES = upper esophageal sphincter.

**Figure 1.**
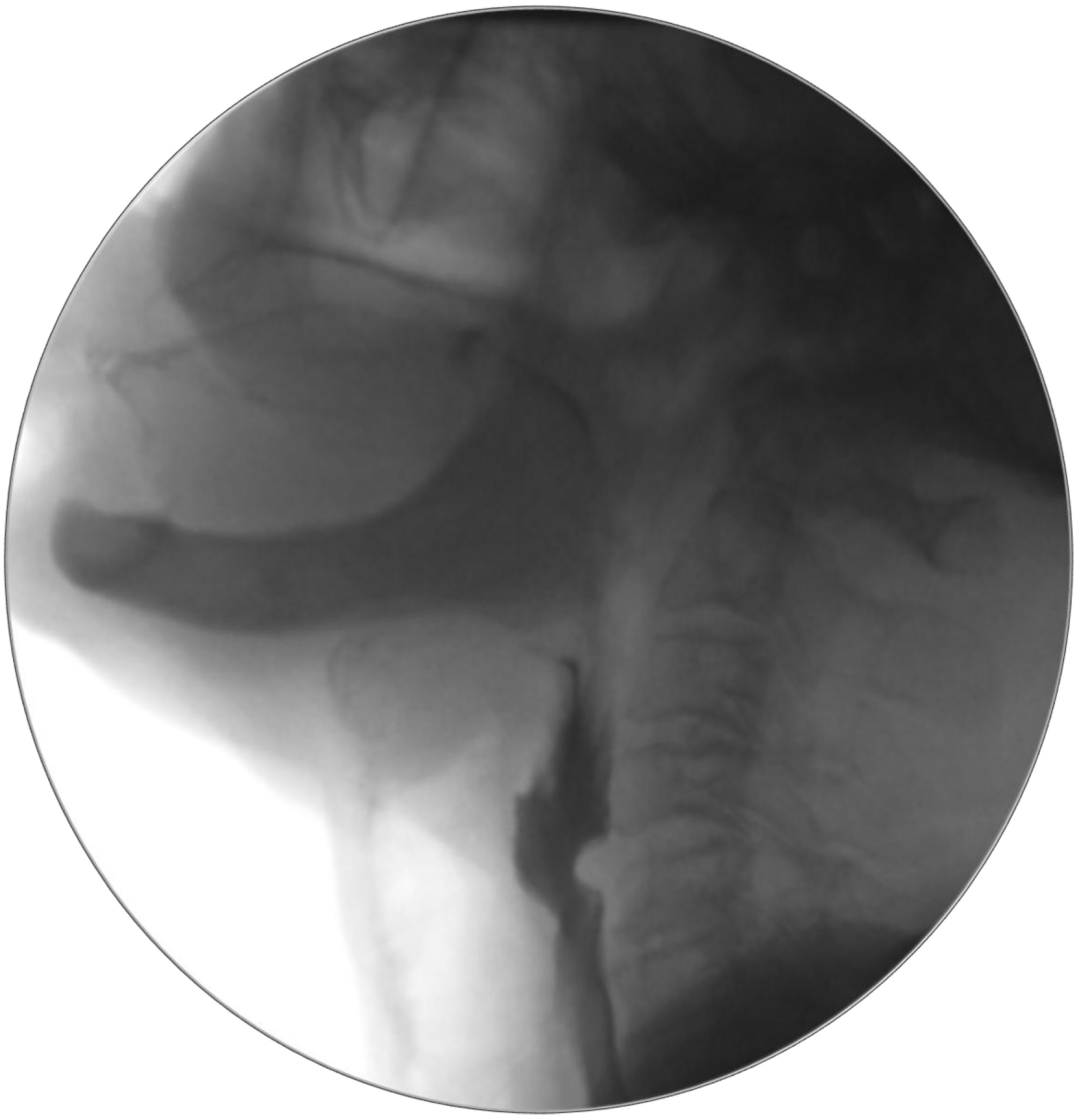
Example videofluoroscopy image showing redundant cricopharyngeal muscle tissue projecting into the pharyngeal lumen during bolus passage.

To further characterize swallowing post-OPHLIIa, the raters documented two additional parameters beyond those previously described. These novel parameters were developed in alignment with ASPEKT method procedures for identifying key frames and performing pixel-based area measurements (Steele et al., 2023) and included:

- A dichotomous categorical parameter to indicate whether or not tissue protrudes into the pharyngeal lumen at the level of the UES during bolus passage (“cricopharyngeal muscle redundancy”; see Figure 1). This phenomenon has the appearance of a cricopharyngeal bar but represents an immobile cricopharyngeus (CP) muscle following surgical severing of CP muscle connections to anterior structures.
- A pixel-based measure of the pyriform sinus area at rest (“hypopharyngeal area”).

### Statistical Analysis

Descriptive statistics were calculated for all continuous parameters. The frequency (%) of atypical values was also calculated for each parameter. Odds ratios for atypical findings were computed using the MedCalc Odds Ratio Calculator (https://www.medcalc.org/calc/odds). Odds ratios were considered statistically significant if their lower 95% confidence interval exceeded 1. For odds ratio calculations regarding impaired swallowing safety, prevalence data from Steele et al. (2023) were used to define reference frequencies of 8% for dichotomized PAS scores of clinical concern and 4% for partial/incomplete LVC. A 25% reference frequency of atypical findings was used for all other parameters with available reference values (Steele et al., 2023). For cricopharyngeal muscle redundancy, a hypothetical reference prevalence of 1% was imputed. Odds ratios could not be calculated for hypopharyngeal area given the absence of healthy reference values for this novel parameter. To compare the OPHLIIa data with healthy reference values, plots were created showing the median and IQR for each quantitative parameter. One-sample *t* tests assessed the magnitude of differences between the OPHLIIa sample values and healthy reference values (Steele et al., 2023). Effect sizes were calculated to quantify group differences, using Cohen's *d* guidelines: values < 0.2 were deemed negligible, 0.2–0.5 small, 0.5–0.8 medium, and > 0.8 large (Kotrlik & Williams, 2003).

## Results

This cross-sectional study involved analysis of archived data from 100 patients (94 men and six women; *M*_age_ = 67 years, range: 28–86 years) who met the inclusion criteria. Available sociodemographic and clinical information regarding these patients is summarized in Table 3. Surgery dates went back as far as 1996. On average, the VFSS exams had taken place 44 months postsurgery (range: 2–219 months). With regard to the distribution of postoperative timing, 16% of the study participants were assessed within the first year, 17% between 12 and 24 months postsurgery, and the remaining 67% more than 2 years postoperatively. This highlights heterogeneity in postoperative timing. Additionally, a subset of participants may still have been within a potential recovery window. At the time of VFSS, four patients had a tracheostomy tube in place, and no patients were assessed with a nasogastric feeding tube. It is not known how many patients had gastrostomy tubes at the time of the VFSS or what their dietary status was at that time.

**Table 3.** Sociodemographic and clinical baseline characteristics (*n* = 100).

Characteristic	Subcategory	Unit	Mean (range) or frequency
Age		Years	67 (28–86)
Time postsurgery		Months	44 (2–219)
Sex	Male	%	94
Preserved arytenoids	Both	%	76
	One	%	24
Reported alcohol consumption	Yes	%	56
	No	%	27
	Ex-drinker	%	11
	Not reported	%	6
Reported smoking status	Yes	%	51
	No	%	15
	Ex-smoker	%	29
	Not reported	%	5
Cancer stage	I	%	9
	II	%	32
	III	%	45
	IV	%	7
	Not reported	%	7
Comorbidities	Chronic obstructive pulmonary disease	%	29
	Diabetes mellitus	%	9
	Systemic arterial hypertension	%	32
Radiotherapy	No	%	67
	Adjuvant	%	27
	Neoadjuvant	%	2
	Not reported	%	4
Chemotherapy	No	%	92
	Yes	%	3
	Not reported	%	5
Tracheostomy	No	%	6
	Yes	%	87
	Not reported	%	7
Time to decannulation		Days	38 (6–760)
Alternative feeding routes	Nasogastric tube	%	93
	Percutaneous endoscopic gastrostomy tube	%	0
	Not reported	%	7
Time to feeding-tube removal		Days	44 (12–504)

Across raters, preconsensus absolute agreement for PAS scores (full scale) was seen for 94/100 ratings. The remaining six ratings involved two cases where one rater assigned a score of 1, while the other assigned scores of 5 or 6, and four cases where one rater assigned a score of 3 and the other assigned a score of 8. When PAS was dichotomized into scores of concern (3, 5, 6, 7, or 8) versus no concern (1, 2, or 4), almost perfect preconsensus agreement was seen (κ = .88). Agreement was also strong for dichotomized ratings of LVC integrity (complete vs. partial/incomplete: κ = .72). Regarding event identification, the median absolute difference between raters was three frames (IQR = 1–6 frames). ICCs for event identification were .99 (95% confidence interval [.98, .99]). For pixel-based measures, the median absolute preconsensus difference across raters measured 0.1% (C2–C4)^2^, with an IQR from 0.02% to 0.36%. ICCs for pixel-based measures were 1.0 (95% confidence interval [.98, 1.0]).

Descriptive statistics and *t*-test results comparing values in the OPHLIIa sample to healthy reference values for all continuous parameters are shown in Table 4. Frequencies and odds ratios for atypical values are shown for categorical parameters in Table 5 and for continuous parameters in Table 6. Compared to reference frequencies in healthy adults, the OPHLIIa patients exhibited significantly higher odds of atypical findings for PAS scores of concern, partial/incomplete LVC, cricopharyngeal muscle redundancy, prolonged hyoid-burst-to-UESO interval, reduced peak XY hyoid position and hyoid XY speed, increased pharyngeal area at maximum constriction (indicating poor constriction), and residue in all pharyngeal areas.

**Table 4.** Descriptive statistics for continuous parameters.

Parameter	Units	*M*	*SD*	25th percentile	*Mdn*	75th percentile	Healthy reference median^a^	Proposed definition for typical values^a^	*t* test result (*df*)	*p* value	Cohen's *d*
Swallow reaction time	ms	99	297	−33	33	198	133	≤ 400	−0.56 (98)	n.s.	−0.06
Hyoid-burst-to-UES-opening interval	ms	165	165	99	132	198	100	≤ 133	3.98 (98)	< .001	0.4
UES opening duration	ms	528	198	429	495	594	467	≥ 434	3.31 (99)	< .001	0.33
Time-to-most-complete LVC	ms	−297	330	−495	−231	−33	133	≤ 167	−12.4 (97)	< .001	−1.5
LVC duration	ms	1320	990	693	1073	1526	467	≥ 400	8.35 (90)	< .001	0.88
Peak XY hyoid position	%(C2–C4)	145	22	128	144	156	172	≥ 163	−12.84 (99)	< .001	−1.28
Hyoid speed	%(C2–C4)/s	90	38	61	99	117	122	≥ 103	−8.51 (99)	< .001	−0.85
Maximum UES distention	%(C2–C4)	26	8	20	25	31	21	≥ 17	6.46 (99)	< .001	0.65
Pharyngeal area at rest	%(C2–C4)^2^	58.11	23.68	45.15	56.0	72.99	59	≥ 51 and ≤ 70	−.37 (98)	n.s.	−0.04
Hypopharyngeal area at rest	%(C2–C4)^2^	5.8	4.08	3.0	4.56	7.48	N/A	N/A	N/A	N/A	N/A
PhAMPC	%(C2–C4)^2^	23.8	14.5	12.72	19.71	33.54	1.00	≤ 2.7	15.5 (96)	< .001	1.57
Vallecular residue	%(C2–C4)^2^	1.68	2.5	0.0	0.43	2.58	0.10	≤ 0.7	6.3 (98)	< .001	0.63
Pyriform sinus residue	%(C2–C4)^2^	2.89	4.03	0.0	1.88	4.22	0.00	≤ 0.5	7.15 (98)	< .001	0.72
Other pharyngeal Residue	%(C2–C4)^2^	3.04	5.15	0.00	0.86	3.73	0.00	≤ 0.3	5.87 (98)	< .001	0.59
Total pharyngeal Residue	%(C2–C4)^2^	7.61	7.96	1.55	5.39	11.27	0.20	≤ 1.7	9.27 (98)	< .001	0.93

*Note.* UES = upper esophageal sphincter; LVC = laryngeal vestibule closure; PhAMPC = pharyngeal area at maximum pharyngeal constriction; N/A = not available; n.s. = nonsignificant.

a Data from Steele et al. (2023).

**Table 5.** Frequencies and odds ratios for atypical values for categorical parameters.

Parameter	Score	OPHLIIa frequency	Dichotomized rating	Frequency	Reference frequency	Odds ratio	95% CI	*p* value
Penetration–aspiration scale	1	67%	No concern	74%	92%^a^	3.42	[1.44, 8.12]	< .01
	2	5%						
	4	2%						
	3	3%	Concern	22%	8%^a^			
	5	0%						
	6	4%						
	7	0%						
	8	15%						
LVC integrity	Complete	66%	Typical	66%	96%^a^	12.36	[4.19, 36.5]	< .0001
	Partial	23%	Atypical	34%	4%^a^			
	Incomplete	11%						
Cricopharyngeal muscle redundancy	Absent	61%	Typical	61%	99%	63.29	[8.48, 472.6]	< .001
	Present	39%	Atypical	39%	1%			

*Note.* OPHLIIa = open partial horizontal laryngectomy Type IIa; CI = confidence interval.

a Data from Steele et al. (2023).

**Table 6.** Frequencies and odds ratios for atypical values for continuous parameters.

Parameter	Direction of concern	OPHLIIa frequency	Reference frequency^a^	Odds ratio	95% CI	*p* value
Swallow reaction time	Prolonged	10%	25%	0.33	[0.15, 0.74]	n.s.
Hyoid-burst-to-UES-opening interval	Prolonged	45%	25%	2.45	[1.35, 4.47]	< .005
UES opening duration	Short	33%	25%	1.5	[0.81, 2.78]	n.s.
Time-to-LVC	Prolonged	2%	25%	0.06	[0.01, 0.27]	n.s.
LVC duration	Short	1%	25%	0.03	[0.004, 0.23]	n.s.
Peak XY hyoid position	Lower	83%	25%	14.65	[7.34, 29.22]	< .0001
Hyoid XY speed	Slower	59%	25%	4.75	[2.36, 7.89]	< .0001
Pharyngeal area at rest	Small	37%	25%	1.76	[0.96, 3.24]	n.s.
	Large	29%	25%	1.22	[0.66, 2.29]	n.s.
Maximum UES distention	Narrow	8%	25%	0.26	[0.11, 0.61]	n.s.
PhAMPC	Large	98%	25%	147	[33.75, 640.2]	< .0001
Vallecular residue	Greater	49%	25%	2.88	[1.58, 5.25]	< .001
Pyriform sinus residue	Greater	63%	25%	5.11	[2.78, 9.38]	< .0001
Other pharyngeal residue	Greater	57%	25%	2.1	[2.18, 7.26]	< .0001
Total pharyngeal residue	Greater	76%	25%	9.5	[4.99, 18.1]	< .0001

*Note.* OPHLIIa = open partial horizontal laryngectomy Type IIa; CI = confidence interval; n.s. = not significant; UES = upper esophageal sphincter; LVC = laryngeal vestibule closure; PhAMPC = pharyngeal area at maximum pharyngeal constriction.

a As defined in Steele et al. (2023).

**Figure 2.**
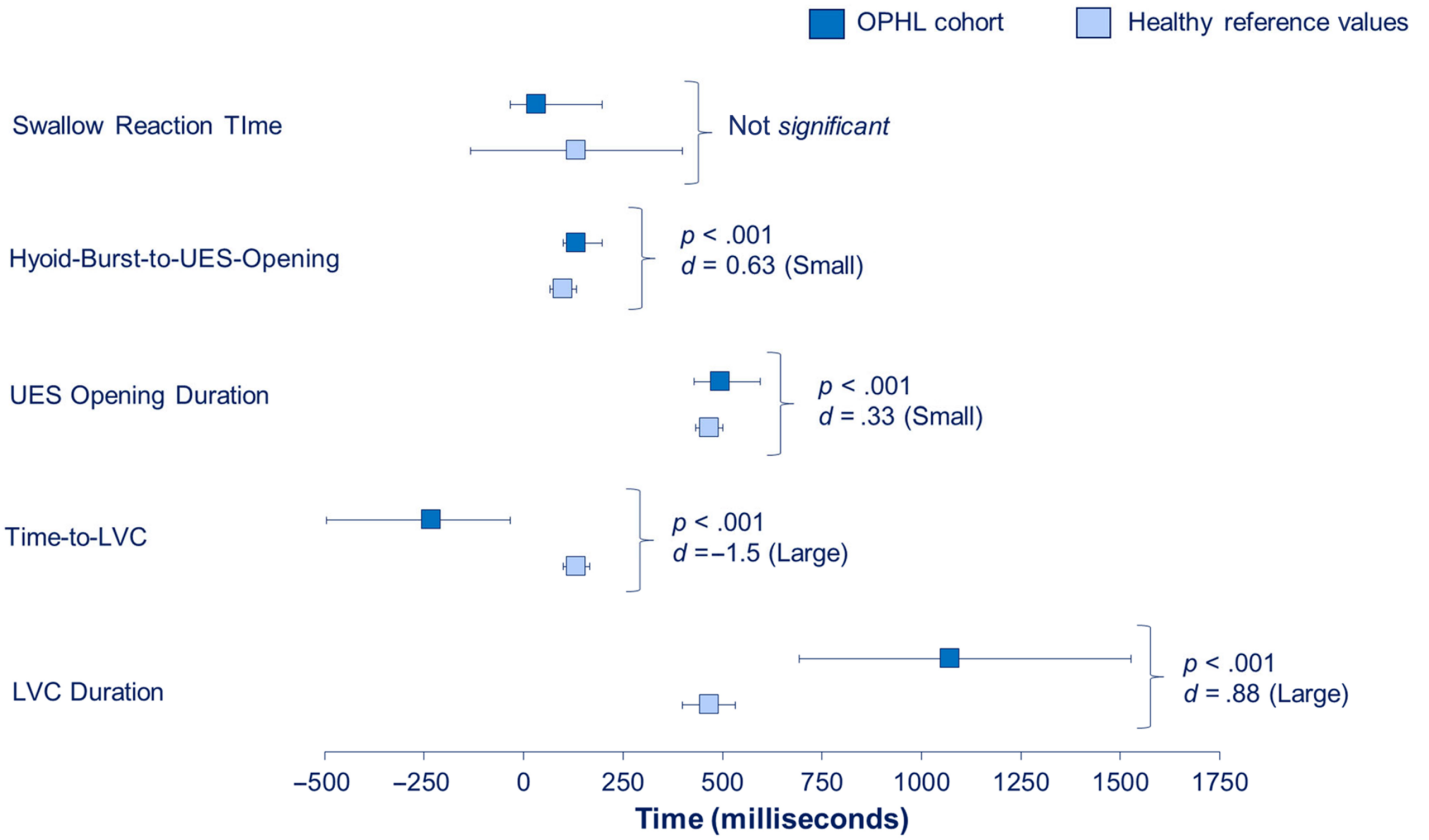
Comparison of medians and interquartile range for timing measures between open partial horizontal laryngectomy Type IIa (OPHLIIa) patients and healthy reference values. LVC = laryngeal vestibule closure; OPHL = open partial horizontal laryngectomy; UES = upper esophageal sphincter.

As illustrated in Figure 2 and Table 4, one-sample *t* tests confirmed significant prolongation of the hyoid-burst-to-UESO interval compared to healthy reference values (small effect). The *t* tests also revealed a significantly longer UESO duration (small effect) and large effects for a significantly shorter time-to-LVC and longer LVC duration, all of which represent potentially protective compensatory mechanisms (see Figure 2).

**Figure 3.**
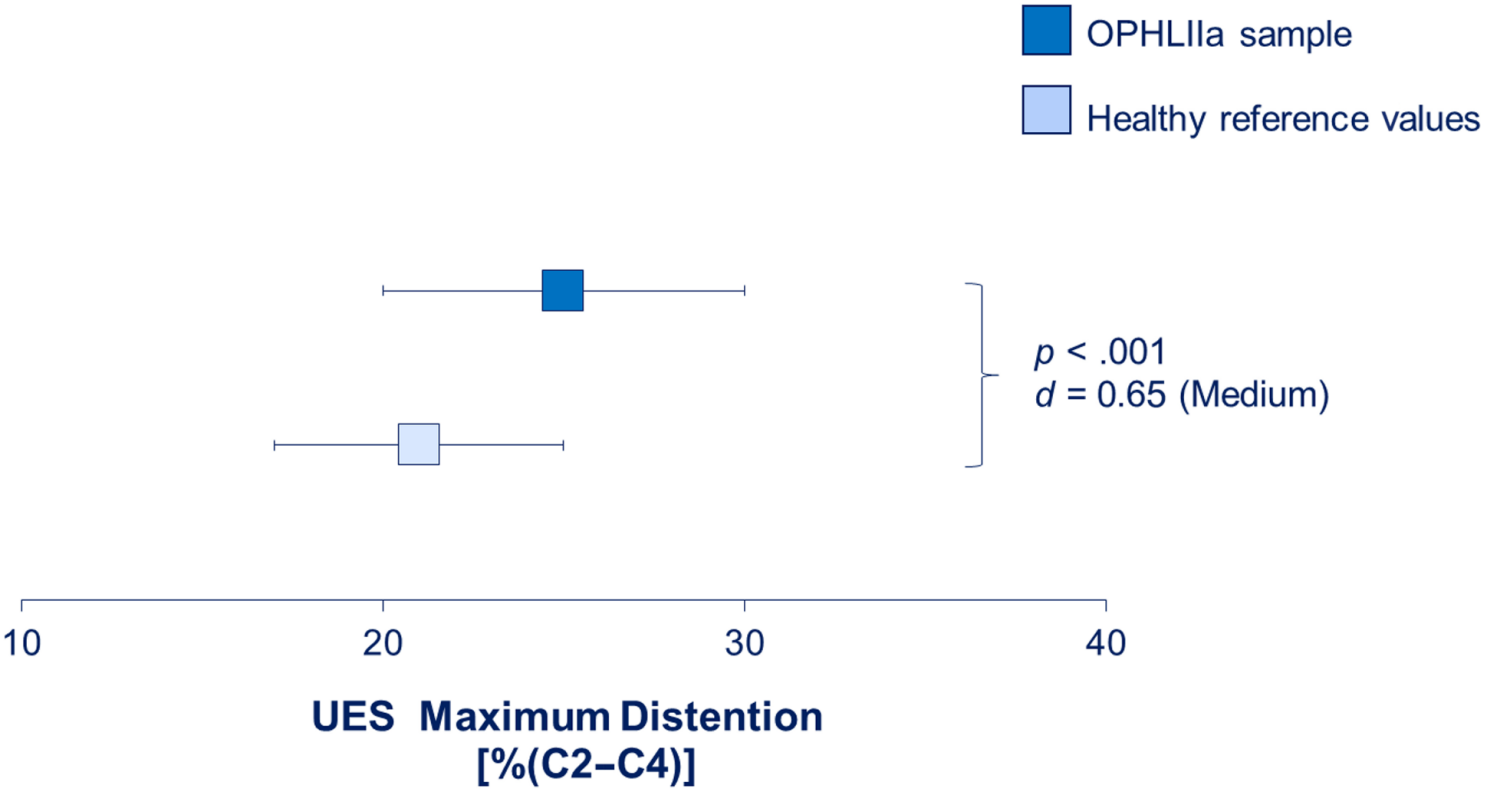
Comparison of medians and interquartile range for upper esophageal sphincter (UES) maximum distention between open partial horizontal laryngectomy Type IIa (OPHLIIa) patients and healthy reference values.

**Figure 4.**
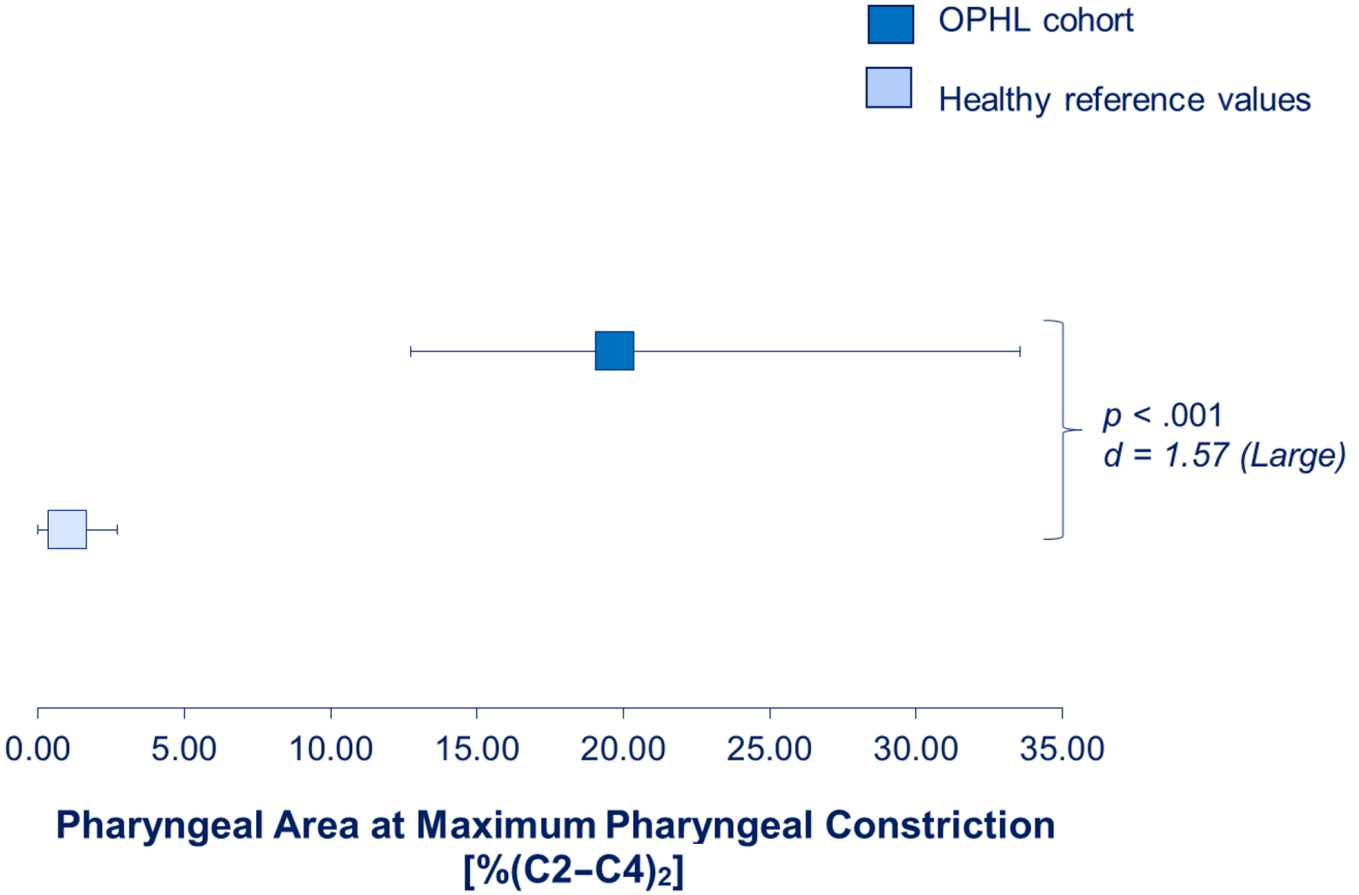
Comparison of medians and interquartile range for maximum pharyngeal constriction between open partial horizontal laryngectomy (OPHL) Type IIa (OPHLIIa) patients and healthy reference values.

**Figure 5.**
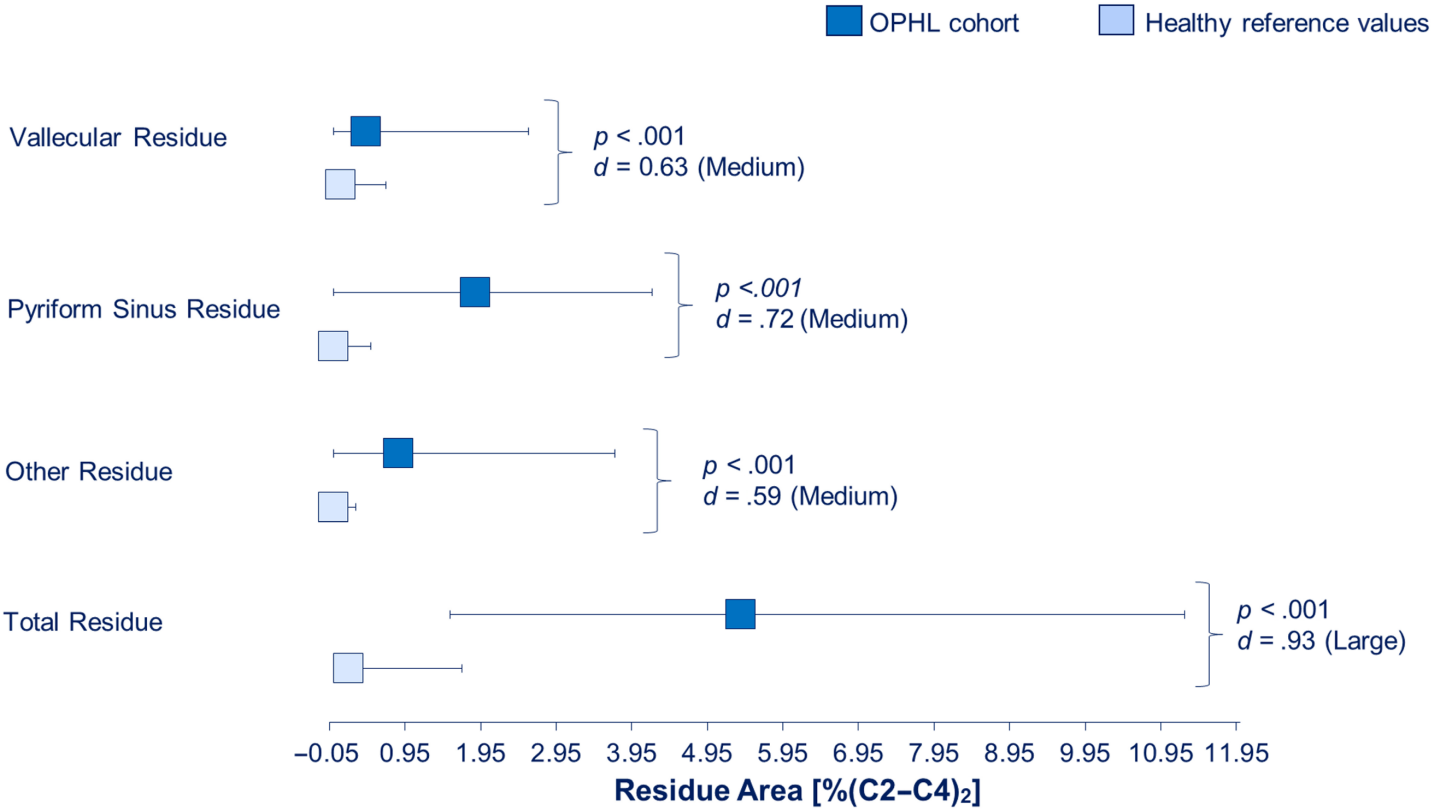
Comparison of medians and interquartile range for residue between open partial horizontal laryngectomy (OPHL) Type IIa (OPHLIIa) patients and healthy reference values.

With regard to kinematics and efficiency, the one-sample *t* tests revealed significantly reduced peak hyoid position and hyoid speed, both with large effect size. Significantly increased maximum UES distention (medium effect size; see Figure 3) and increased pharyngeal area at maximum constriction (large effect size; see Figure 4) were also seen. Consistent with observations reported in our previous pilot study (Barbosa et al., 2024), particularly poor pharyngeal constriction was noted in the hypopharynx. Median area measurements for the hypopharyngeal area at rest were 5.8% (C2–C4)^2^ (interquartile range = 5%–6.6%). Significantly greater residue in all locations (medium-to-large effect sizes) was also found compared to reference values (see Figure 5). Cricopharyngeal muscle redundancy was observed in 39% of the participants. Despite this, only 8% of the sample showed atypically narrow UESO.

## Discussion

The OPHLIIa technique involves removal of multiple laryngeal structures, the loss of anterior mucosal and muscular connections to the inferior pharyngeal constrictor and CP muscles, and the surgical repositioning of the cricoid cartilage toward the hyoid bone. CHEP impacts the positioning, dimensions, and attachments of nearby structures, including the pyriform sinuses and the inferior pharyngeal constrictor muscle. These anatomical modifications impact swallowing safety, efficiency, and biomechanics. This study provides a comprehensive quantitative analysis of altered swallowing function and physiology after OPHLIIa, through the comparison of measures obtained using the ASPEKT method to healthy reference values (Steele et al., 2023), advancing our understanding of changes in swallowing following this procedure.

The findings corroborate previous reports of impaired LVC in this population (Goeleven et al., 2005; Kelchner & Klaben, 2013; Lewin et al., 2008; Liu et al., 2024; Pizzorni et al., 2019; Schindler, Pizzorni, Fantini, et al., 2016), accompanied by 3.42-fold increased odds of PAS scores of clinical concern. Notably, and consistent with prior reports, when aspiration into the trachea occurred, it was silent (Woisard et al., 1996; Zacharek et al., 2001). The findings also concur with previous reports of inefficiency and residue as prominent characteristics of swallowing after OPHLIIa (Barbosa et al., 2024; Lewin et al., 2008; Pizzorni et al., 2019; Schindler, Pizzorni, Fantini, et al., 2016). With regard to mechanisms underlying residue, poor pharyngeal constriction was noted to be an extremely frequent finding, consistent with previous reports of poor tongue base retraction in these patients (Lewin et al., 2008; Pizzorni et al., 2019). Observations of particularly poor constriction of the hypopharynx and a high frequency of cricopharyngeal muscle redundancy suggest additional changes due to altered muscular attachments, loss of structural support, and possible denervation of the inferior pharyngeal constrictor and CP muscles. These factors likely contribute to residue accumulation after OPHLIIa.

Furthermore, the study results confirm several additional observations from our previous pilot study (Barbosa et al., 2024) including a prolonged hyoid-burst-to-UESO-interval, increased maximum UES distention, and prolonged durations of LVC and UESO. Findings from the Barbosa et al. (2024) study that were not seen in this larger sample include prolonged time-to-LVC (the opposite finding was observed) and reduced pharyngeal area at rest. New findings in the current analysis, which were not reported in Barbosa et al. (2024), include significant reductions in hyoid peak XY position and hyoid speed. Previous studies have attributed reduced hyoid excursion after OPHLIIa to altered cricohyoid linkages and surgical scarring (Lewin et al., 2008; Pizzorni et al., 2019).

In our previous pilot study (Barbosa et al., 2024), we noted that some of these observed changes suggested compensations rather than impairments of swallowing physiology. Evidence of such compensations is substantiated in the current analysis and includes shortened time-to-LVC and prolonged LVC duration to support airway protection, wider UES distention, and prolonged UESO duration to facilitate greater bolus clearance and a low frequency of atypically narrow UESO, despite a high frequency of redundant cricopharyngeal tissue. These observations are consistent with prior reports that compensatory strategies can be effective in mitigating the risk of aspiration after partial laryngectomy (Lewin et al., 2008; Pizzorni et al., 2019; Woisard et al., 1996). In the current sample, it is unknown to what extent the apparent compensations reflect spontaneous or learned techniques to improve swallowing. However, the fact that several of these findings were characterized by large effect sizes highlights their critical role as protective strategies that help to sustain functional swallowing despite structural alterations after OPHLIIa.

The impairments observed in this study underscore the necessity for targeted rehabilitation strategies addressing both swallowing safety and efficiency after OPHLIIa. Effective therapeutic approaches might include a focus on use of instructed compensations to extend LVC and UESO duration. Strategies such as the supraglottic swallow and the effortful swallow should also be explored for their benefits in reducing aspiration and residue in this population (Lewin et al., 2008; Pizzorni et al., 2019). Given the retrospective nature of the study and the absence of detailed data regarding rehabilitation type, intensity, and adherence, conclusions regarding the adequacy or effectiveness of specific swallowing exercises or interventions cannot be drawn from this study's findings.

### Limitations and Future Directions

Although this study contributes robust quantitative data based on a large sample of OPHLIIa patients, several limitations must be acknowledged. Foremost among these is the fact that the analysis was performed on an archived data set, limiting our ability to explore additional information about the study participants or to consider stratification of the data. Given the extended time frame of the surgical procedures (1996–2018), it is highly probable that multiple surgeons performed the OPHLIIa surgeries. While the core OPHLIIa technique involves removal of specific anatomical structures (pre-epiglottic space, epiglottic pedicle, thyroid cartilage, laryngeal ventricles, vocal folds, vestibular folds, and paraglottic space, with potential preservation of one or both arytenoids), individual surgeon variations may have occurred but were not systematically recorded in the available data set fields.

Another significant limitation is the wide variability in the time interval between surgery and VFSS. As a result, our data reflect swallowing physiology at different long-term postoperative stages rather than at standardized follow-up time points. This lack of uniformity in postsurgical assessment timing potentially introduces significant variability in the data. For future studies, we encourage implementation of a longitudinal design with several fixed VFSS assessment points to inform better understanding of functional recovery after OPHLIIa. Given the long study time frame, protocol variations were also present in the archived VFSS recordings that were available for secondary analysis. For this reason, the current analysis was limited to ratings of a single thin liquid bolus per patient, which may underrepresent variability in swallowing. This not only limits the generalizability of the results but means that the study findings do not allow us to understand how variations in bolus volume or consistency might impact swallowing after OPHLIIa or to envision that the features identified in this study might manifest in longer and more comprehensive VFSS protocols, such as those used for the Modified Barium Swallow Impairment Profile (Martin-Harris et al., 2008) or Dynamic Imaging Grade of Swallowing Toxicity (Hutcheson et al., 2017, 2022). We also acknowledge that although the comfortable thin liquid sip task in these archived VFSS recordings overlaps with the tasks used to develop ASPEKT reference values (Steele et al., 2023), it was collected using a cued swallow technique, which differs from ASPEKT methodology. This methodological difference had potential to lead to shorter swallow reaction time measures in the OPHLIIa sample (Daniels et al., 2007; Nagy et al., 2013). The fact that this was not observed suggests that this particular methodological difference was of negligible impact. Nevertheless, future prospective studies should pay close attention to protocol and instructional details to maximize comparability across rating methods.

## Conclusions

This study is a secondary analysis of archived cross-sectional VFSS data for patients who had undergone OPHLIIa surgery. Detailed analysis of quantitative VFSS metrics for recordings for sips of thin liquid reveals significant alterations in swallowing function and physiology, including compromised LVC integrity, poor pharyngeal constriction, reduced hyoid movement, and increased residue. The data also corroborate previous evidence of compensations in the timing and duration of LVC and the duration and extent of UESO, which may serve to facilitate improved swallowing safety and efficiency in this sample. The study highlights poor pharyngeal constriction, particularly in the hypopharynx, as a defining characteristic of this postsurgical population. These findings highlight the importance of tailored rehabilitation strategies to improve swallowing efficiency and safety in affected individuals. Future longitudinal studies are needed to document the progression of swallowing function and the efficacy of targeted rehabilitation in this population.

## Data Availability Statement

The data sets generated during and/or analyzed during the current study are not publicly available due to ethical/legal restrictions. Inquiries regarding access to the data should be directed to the corresponding author.
